# Pterisolic Acid B is a Nrf2 Activator by Targeting C171 within Keap1-BTB Domain

**DOI:** 10.1038/srep19231

**Published:** 2016-01-13

**Authors:** Ting Dong, Weilong Liu, Zhirong Shen, Lin Li, She Chen, Xiaoguang Lei

**Affiliations:** 1 Beijing National Laboratory for Molecular Sciences, Key Laboratory of Bioorganic Chemistry and Molecular Engineering of Ministry of Education, Department of Chemical Biology, College of Chemistry and Molecular Engineering, Synthetic and Functional Biomolecules Center, and Peking-Tsinghua Center for Life Sciences, Peking University, Beijing 100871 (China); 2 National Institute of Biological Sciences (NIBS), Changping District, Beijing 102206 (China)

## Abstract

The use of chemoprotective agents to minimize the side effects of the chemotherapy, primarily via activation of the Nrf2 pathway, is an emerging research field, which has attracted broad attention from both academia and pharmaceutical industry. Through high-throughput chemical screens we have disclosed that pterisolic acid B (**J19**), a naturally occuring diterpenoid, is an effective Nrf2 activator. We have also identified a more potent natural product analogue **J19-1** by semisynthesis and the subsequent biochemical evaluations revealed that **J19-1** activates the Nrf2 pathway by covalently modifying Cys171 of keap1, which inhibits Nrf2 degradation mediated by Keap1-Cul3 complexes. Ultimately, we have demonstrated that **J19-1** shows significant cytoprotective effect against cisplatin-induced cytotoxicity in HKC cells.

The landscape of cancer treatment is changing dramatically. Traditional chemotherapeutics, which have dominated treatment for over half a century, are typically DNA-damaging or microtubule-targeting agents designed to inhibit or kill rapidly dividing cells^1^,^2^,^3^,^4^. However, severe side effects, such as organ damage, nausea and hair loss are often encountered because chemotherapeutic drugs cannot distinguish between cancer and rapidly proliferating healthy cells. Side effects may be reduced by targeted therapy which selectively acts upon mutations within cancer cells^5^, or by the administration of chemoprotective drugs, generally dietary or synthetic anti-oxidants^6^,^7^,^8^.

Current studies suggest that chemoprotective drugs accelerate the metabolism and excretion of toxic chemotherapy agents within specific tissues by the induction of phase II detoxification enzymes. Enzymes such as glutamate cysteine ligase (GCS) and quinone oxidoreductase-1 (NQO1)^9^,^10^,^11^, as well as NAD(P)H are involved in glutathione synthesis, elimination of reactive oxygen species (ROS), xenobiotic metabolism and drug transport^12^. The Nrf2 pathway is an essential cellular system to protect tissues from environmental stress. In general, expression of protective enzymes is induced by binding of the Nrf2 transcription factor to the antioxidant responsive element (ARE) located in the enhancer sequence of the genes^13^,^14^. The cytoplasmic level of Nrf2 is regulated by Keap1 (Kelch-like ECH-associated protein 1) and the BTB domain within Keap1 serves as an adaptor that bridges Nrf2 to a Cul3-Based E3 Ligase, finally leading to Nrf2 degradation^15^,^16^.

In view of their pivotal role in cell protection from environmental insults, several Nrf2 activators are currently undergoing clinical trials as cytoprotective agents^17^,^18^,^19^. For example, N-acetylcysteine has been demonstrated to protect the kidney against ischemic injury via regulation of the Nrf2 pathway^20^,^21^. Inspired by these promising results, the development of novel effective Nrf2 activators and elucidation of the molecular mechanisms may provide a great impact to cancer therapy. Here, we describe the discovery and mode of action of a new Nrf2 activator, a naturally occurring diterpenoid, pterisolic acid B (**J19**), through high-throughput chemical screens using two robust screening systems as well as subsequent biochemical studies. We also demonstrate that pterisolic acid B has significant cytoprotective effect for normal tissue cells in cancer chemotherapy in a Nrf2 dependent manner.

## Results

**Figure 1:**
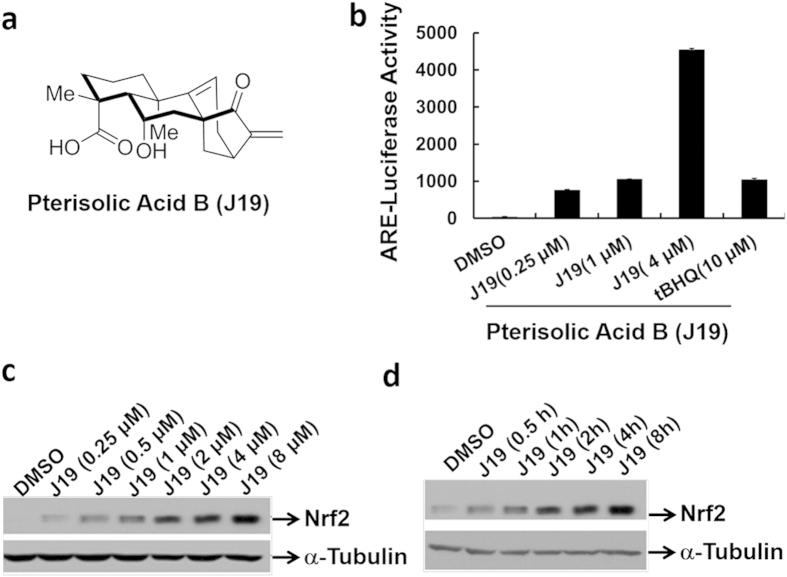
Pterisolic acid B (J19) was validated to activate ARE-luciferase activity in a dose-dependent manner in the MDA-MB-231-ARE-Luc stable cell line. (**a**) Structures of **J19**. (**b**) MDA-MB-231-ARE-Luc stable cells were treated with **J19** at indicated concentrations for 24 h, and subject to luciferase assay. Error bars indicate the standard deviations from triplicate samples. *tert*-Butylhydroquinone (tBHQ), a highly effective antioxidant was used as a positive control. (**c**) HKC cells were treated with **J19** at indicated doses for 4.5 h, and total lysates were subject to immunoblot analysis with anti-Nrf2 and anti-α−Tubulin antibodies. (**b**) HKC cells were treated with 4 μΜ of **J19** for the indicated time points, then the total lysates were subject to immunoblot analysis with anti-Nrf2 and anti-α -Tubulin antibodies.

With an aim to discover novel Nrf2 activators as lead compounds for the development of chemoprotective agents, we used two established robust cell-based high-throughput screening systems: 1) the canonical ARE-driven assay reports compounds which activate the Nrf2 downstream gene ARE in MDA-MB-231 cells; 2) the Neh2-driven reporter system, designed to select compounds which interrupt the Nrf2 signalling pathway^22^,^23^. This second complementary assay was required because ARE may be activated via other pathways, such as PI3K/Akt^24^,^25^. Applying these two cell-based assays, we efficiently screened a chemical library of ~30,000 small molecules including ~800 structurally diverse natural products. We envisioned that the positive compounds from both assays would be of particular interest. After the primary screen, we identified 26 different hits from both assays. Then we performed the secondary validation screen by changing various concentrations. In the end, we identified an interesting natural product, a structurally complex diterpenoid, pterisolic acid B (**J19**) which was originally isolated from of the fern *Pteris semipinnata*^26^, as the most promising hit (Fig. 1a). **J19** could significantly activate both ARE-luciferase (Fig. 1b) and Neh2-luciferase (Supplementary Fig. S1) transcription in a MDA-MB-231 stable cell line.

**Figure 2:**
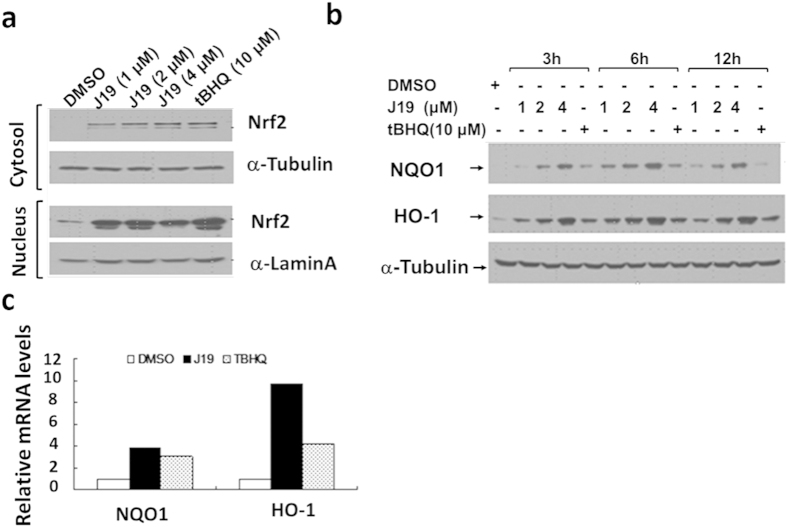
J19 induces activation and nuclear translocation of Nrf2 which subsequently triggers the transcription of downstream protective genes. (**a**) **J19** induces nuclear translocation of Nrf2 in HKC cells. HKC cells were treated with either **J19** or tBHQ for 4.5 h, followed by Western blot analys1is of cytosolic and nuclear fractions. (**b**) **J19** promotes the expression of cytoprotective enzymes. HKC cells were treated with **J19** at various time and subjected to Western blot analysis after HKC cells were treated by the indicated time points and doses. (**c**) HKC cells were treated with 4 μΜ of **J19** for 12 h and subjected to qRT-PCR analysis.

We then investigated the ability of **J19** to stabilize Nrf2 and induce expression of cytoprotective enzymes in HKC cells. From Western blot analysis, we confirmed that **J19** was capable of inducing Nrf2 activation in both dose- and time- dependent manner (Fig. 1c,d). Notably, we also observed that **J19** increases Nrf2 nuclear translocation (Fig. 2a) and expression of NQO1 and HO-1, which were analyzed by either Western blot (Fig. 2b) or qPCR (Fig. 2c).

**Figure 3:**
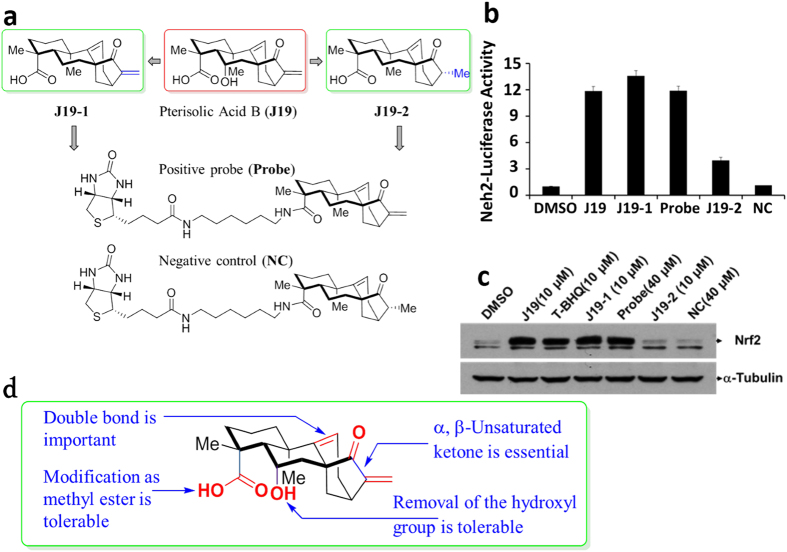
SAR studies of J19 analogs as well as the preparation of positive and negative chemical probes. (**a**) Structures of **J19** and its analogs and Probes (**b**) N23 cells were treated with 4 μΜ of **J19** and its analogues for 12 h and then subject to Luciferase assay. Error bars indicate the standard deviations from triplicate samples. (**c**) Western blot analysis after HKC cells treated by **J19** and its analogues for 4.5 h using the indicated antibodies. (**d**) SAR studies of **J19** analogs.

In order to develop an effective chemical probe for target ID to dissect the mechanism underlying the **J19**-induced Nrf2 activation, we first explored the preliminary structure-activity relationships (SARs) of **J19**. Due to the limited availability of this structurally complex natural product **J19** as well as its biosynthetic congeners, we were not able to make a large number of analogs to fully address the SARs. Fortunately, a promising natural product analog **J19-1** (Fig. 3a) was identified through the biological evaluations of a small focused library of our synthesized compounds using the Neh2-luciferase assay. Remarkably, **J19-1** was proven to be more potent to activate Nrf2 than the parent natural product **J19** (Fig. 3b,c). Interestingly, we also observed that the analog **J19-2** containing a saturated enone moiety lost the activities significantly (Fig. 3b,c). Based on the SARs result (Fig. 3d), which suggested that **J19** might covalently bind to its target protein via a cysteine Michael addition. The above-mentioned studies eventually enabled us to prepare an effective chemical probe (**Probe**) as well as a negative control compound (**NC**) from **J19-1** and **J19-2** respectively (Supplementary Information).

**Figure 4:**
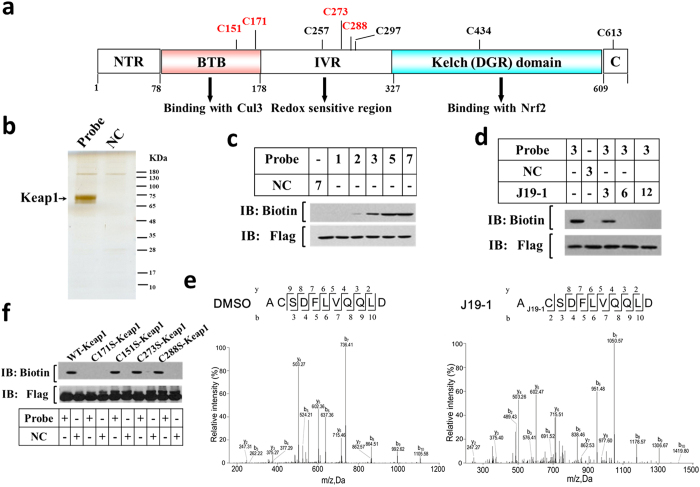
J19-1 directly targets Keap1 at C171. (**a**) Domain structures of human Keap1. Locations of all cysteines and important binding partners are shown, and four key cysteines (C151, C171,C273, and C288) are highlighted in red (human Keap1). (**b**) Total lysates of Keap1-Flag transfected 293 T cells were incubated with **Probe** or **NC** at 4 °C overnight. The precipitates resolved by SDS-PAGE were stained by silver staining. (**c**) The recombinant Keap1 protein was incubated with **Probe** or **NC** at 37 °C for 1.5 h, and the mixtures were blotted for biotin or Keap1. (**d**) The recombinant Keap1 protein were incubated with **Probe** in the absence or presence of a 2–3 fold excess of unlabeled **J19-1** for 1.5 h at 37 °C, and the mixtures were blotted for biotin or Flag. (**e**) MS/MS analysis of the recombinant Keap1 incubated with or without **J19-1** for 1.5 h. The red arrow indicates the cysteine bound to **J19-1**. (**f**) Recombinant wild-type (WT) Keap1 and its mutants were incubated with Probe or NC at 37 °C for 1.5 h, followed by blotting for biotin and Keap1.

Since the SAR studies revealed the essential role of the α, β-unsaturated ketone moiety of **J19-1**, which could serve as a Michael acceptor to form a covalent bond with a cysteine residue of its interacting protein. Keap1, a cytosolic repressor of Nrf2, has been reported to possess 27 cysteine residues and some of them are highly reactive (Fig. 4a)^27^,^28^,^29^,^30^. To determine whether **J19-1** targeting Keap1 through thiol modification activates Keap1-Nrf2 signaling pathway, we therefore synthesized the **Probe** and **NC** and examined their bioactivity. As shown in Fig. 3b,c, **Probe** retained the ability to effectively induce Nrf2 activation at 4 μM while the NC was completely inactive at the same concentration. Encouraged by this result, target identification with both **Probe** and **NC** was performed using affinity pull-down experiments. Keap1-Flag transfected 293 T cell lysates were incubated with streptavidin agarose beads which were pre-coupled with **Probe** or **NC**. The proteins precipitated by streptavidin agarose beads were resolved by SDS-PAGE and stained with silver. As shown in Fig. 4b, one clear band was specifically precipitated by **Probe** but not by **NC**. Peptide mass fingerprinting data analysis revealed that the probe-bound protein was Kelch-like ECH-associated protein 1 (Keap1). We validated the result by probing the precipitates with Keap1 antibody (Supplementary Fig. S2). Furthermore, we found that the probe interacted with the recombinant Keap1 in good dose dependent manner (Fig. 4c), and this interaction could be completed off with 2 folds of **J19-1** (Fig. 4d). Collectively, these data supported the conclusion that Keap1 is the cellular target of **J19**.

In order to further identify which residues are critical for the binding process, we incubated DMSO or **J19-1** with HKC cells for 1.5 h and then the harvested proteins for liquid chromatography tandem mass spectrometry (LC/MS/MS) analysis. As shown in Fig. 4e, the chymotryptic peptide containing Cys171 (amino acid 170–180) exhibited a mass increase of 330.18 relative to peptide fragments containing Cys171, indicating that Cys171 is covalently modified by **J19-1**. Consistent with this result, when **J19-1** was incubated with WT or selected recombinant protein mutants *in vitro*, the probe binding band was not observed when cysteine 171 was mutated to serine (Fig. 4f). Among 27 cysteine residues in Keap1, 5 cysteine residues have been reported to be directly modified by oxidative agents^27^,^28^,^29^,^30^. C151 is most reactive toward N-iodoacetyl-N-biotinylhexylenediamine^27^, as well as natural products sulforaphane, isoliquiritigenin, xanthohumol and 10-shogaol^28^,^29^. C171 in KEAP1 is also reported as a mechanism of NRF2 activation^31^.

**Figure 5:**
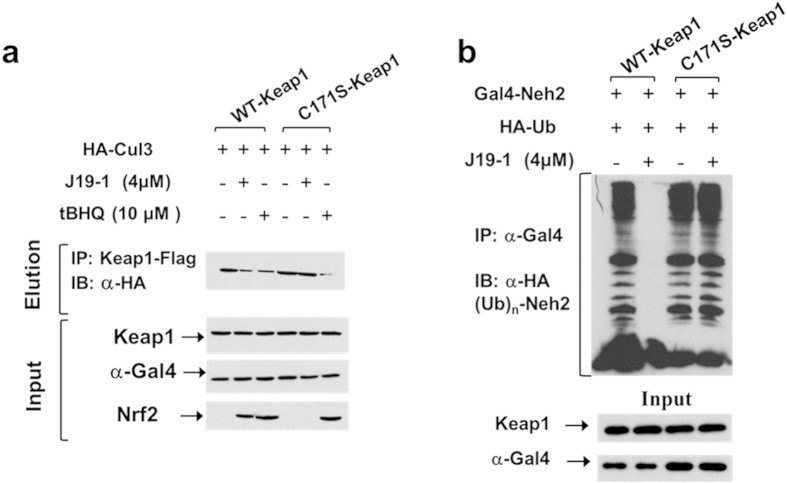
J19-1 actives Nrf2 pathway by inhibiting Keap1-mediated Nrf2 ubiquitination *in vivo* in a C171 dependent manner. (**a**) HEK293 cells were transiently transfected with WT-Keap1 or C171S-Keap1, Gal4-Neh2, and HA-Ub and treated with indicated concentration of **J19-1** or tBHQ for 5 h. Lysates were immunoprecipitated by anti-Gal4 antibody, and ubiquitination was assessed using anti-HA antibody. (**b**) HEK293 cells were transiently transfected with HA-Cul3 and Flag-tagged WT (or C171S) Keap1, and then treated with tBHQ or 4 μM **J19-1** for 5 h. Pull-down using Flag beads and ensuing immunoblot analysis with anti-HA antibody.

We then sought to further elucidate the mechanism of how the modification of C171 leads to Nrf2 activation. C171 is located within the BTB domain of Keap1, which plays key roles in mediating interactions with the Cul3-E3 ubiquitin ligase system^15^,^30^,^32^. There is literature evidence that alkylation of Keap1 by electrophiles results in the dissociation of the Keap1-Cul3 protein-protein interaction responsible for Nrf2 degradation^13^,^14^. We therefore investigated whether **J19-1** perturbed the interaction between Cul3 and Keap1. As shown in Fig. 5a, **J19-1** indeed interrupted the Keap1-Cul3 interaction. Furthermore, when the C171S Keap1 mutant was used instead, this inhibitory effect disappeared. Next we examined whether **J19-1** caused dissociation of Keap1-Cul3 protein-protein interaction might subsquently result in Nrf2 stabilization. Fig. 5b and Supplementary Fig. S3 indicated that 4 μM of **J19-1** efficiently inhibited Keap1-dependent Neh2 domain of Nrf2 ubiquitination and in a C171S mutant of human Keap1, this down-regulation does not occur. All of these results demonstrated that the modification of Keap1-C171 by **J19-1** disrupted the ability of Keap1 to serve as an adaptor for Cul3-ubiquitin E3 ligase complex, thereby leading to Nrf2 stability and activation. Meanwhile we also confirmed that C171S-keap1 behaved identically to the wild-type Keap1 protein in terms of both repression of Nrf2-dependent reporter gene activity under basal culture conditions and increased Nrf2 dependent reporter gene activity following the exposure to **J19-1** (Supplementary Fig. S4)^33^.

**Figure 6:**
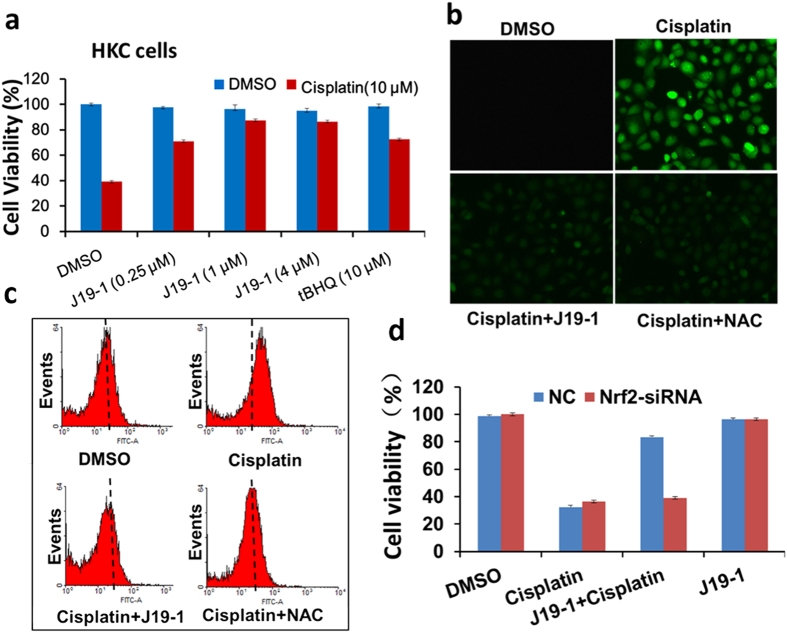
J19-1 protect HKC cells from cisplatin induced cell toxicity by scavenging ROS. (**a**) HKC cells were pretreated with **J19-1** or tBHQ for12 h, and added 10 μΜ of Cisplatin for 24 h, then the cell survival was measured by CCK-8 assay. (**b**,**c**) HKC cells were pretreated with 4 μΜ **J19-1** or 10 mM NAC for 12 h and then added 10 μΜ of Cisplatin for 24 h. The level of intracellular ROS was monitored by using DCFH-DA and analyzed by either fluorescent microscope assay (**b**) or flow cytometry (**c**). ROS scavenger NAC (N-acetyl-L-cysteine) was used as a positive control. (**d**) HKC cells were first transfected with siRNA of Nrf2, then pretreated with 4 μΜ of **J19-1** or tBHQ for12 h, and treated with 10 μΜ of Cisplatin. After 24 h, cell survival was measured by using MTT assay.

After establishing **J19-1** as an effective Nrf2 activator via a new mode of action, we sought to further explore the therapeutic potential of **J19-1** as a chemoprotective agent in cancer chemotherapeutics. Cisplatin is an extensively used chemotherapeutic agent for the treatment of various solid tumors^34^,^35^. However, the severe side effects of cisplatin paticularly including progressive nephrotoxicity caused by increasing production of ROS greatly impair the quality of life^36^. We thus investigated the cytoprotective effect of **J19-1** on cinsplatin-induced normal cell death. Human HKC cells (human kidney cells) were pretreated with 4 μM of **J19-1** for 12 h, followed by exposure to 10 μM of cisplatin for 24 h. Cell viability was determined using MTT method. As shown in Fig. 6a, **J19-1** exhibited dose dependent cytoprotective effects against cisplatin-induced cell death. Furthermore, we also demonstrated that **J19-1** decreased the intracellular ROS level analyzed by both fluorescent microscope assay (Fig. 6b) or flow cytometry (Fig. 6c). Figure 6d showed that the protective effect of **J19-1**was a direct consequence of Nrf2 activation, by using Nrf2 siRNA knockdown. Finally, in order to investigate whether **J19-1** could serve as a good chemo-protective agent, we exmined its effect in antagonizing the therapeutic efficacy of cisplatin in cancer cells, MDA-MB-231 cells. As shown in Supplementary Fig. S5, only slight difference was observed in cell survival between cell treated with cisplatin alone or cotreated with **J19-1**. Meanwhile, we examined the Nrf2 activation level induced by **J19-1** in normal and cancer cells (Supplementary Fig. S6). A549 cells were selected for their somatic mutations driving sustained Nrf2 activation which largely contribute to chemoresistance^37^. As shown in Supplementary Fig. 7, 1 uM of **J19-1** could induce the highest Nrf2 level in HKC cells, but not in in A549 cells. All these results indicated that **J19-1** is a promising chemoprotective agent.

## Discussion

In summary, we have discovered a structurally unique and complex diterpenoid pterisolic acid B (**J19**) as an effective Nrf2 activator through high-throughput chemical screens as well as subsequent biological evaluations. Through target ID and validation, we have elucidated that pterisolic acid B activates the Nrf2 pathway by covalent modification of Cys171 within Keap1, which inhibits Nrf2 degradation mediated by Keap1-Cul3 complexes. Notably, we have further demonstrated that the more potent natural product analog **J19-1** has significant cytoprotective effect against cisplatin-induced cytotoxicity in HKC cells. Pterisolic acid B and its congeners are therefore excellent synthetic targets for the development of novel chemoprotective agents to address the severe side effects of traditional cancer chemotherapeutics.

## Methods

### Cell lines, plasmids and antibodies

The human cells such as MD-MBA 231, HEK293T, HKC, A549 cells were obtained from the ATCC, DMEM medium, phosphate buffered saline (PBS), Trypsin, and Penicillin/streptomycin were purchased from GIBCO. ATP, MgCl2, Fetal bovine serum (FBS), TBHQ, DMSO were purchased from Sigma. Western Lightning® Plus-ECL kit was purchased from PerkinElmer. Cell Counting Kit-8 was purchased from (Dojindo). Protein Assay kit was purchased from Bio-Rad. QuikChange® site-directed mutagenesis kit was from Stratagene.Nuclear and Cytoplasmic Protein Extraction Kit was purchased Sangon Biotech. The oxidant-sensitive probe 2’,7’-dichlorodihyd rofluorescein diacetate (DCHF-DA) was purchased from Sigma-Aldrich. ARE-luciferase reporter plasmid and pGL6 plasmid were purhcased from Beyotime Institute of Biotechnology. Flag-Keap1, C151S-Keap1, C273S-Keap1, C288S-Keap1, Flag-Nrf2, Gal4-Neh2 and Neh2-luciferase reporter plasmid were kindly gifted from Xiaodong Wang’s Lab (NIBS). The plasmids of HA-ubiquitin, HA-Cul3 were purchased from Addgene. The antibodies of Nrf2 (ab76026), keap1 (ab66620) and Lamin A antibody (ab26300) were purchased from Abcam, the antibodies of HA, Gal4, Flag were purchased from Sigma. The antibodies of HO-1 and NQO1 were purchased from Santa Cruz Biotech.

### Generation of stable ARE-driven reporter systems

The ARE-luciferase reporter plasmid, which was purchased from Beyotime Institute of Biotechnology, along with the pGL6 plasmid containing the neomycin selectable marker, was stably transfected into MDA-MB-231 using Lipofectamine 2000 from Invitrogen (Grand Island, NY, USA) according to the manufacturer’s instructions. At 48 h post-transfection, transfected cells were selected using 0.8 mg/mL G418 in the media for 3 to 4 weeks. The G418-resistant clones were isolated and screened by measuring their basal and inducible (obtained by treatment with 10 umol/L tBHQ) luciferase activities as described above. Positive clones, which showed low background and high inducible luciferase activity, were passaged and maintained in growth medium containing 0.8 mg/mL G418.

### Generation of stable Neh2-driven reporter systems

The Neh2-luciferase reporter plasmid, which was kindly provided by Prof. Xiaodong Wang’s Lab, along with the pGL4 plasmid containing the neomycin selectable marker, was stably transfected into MDA-MB-231 using Lipofectamine 2000 from Invitrogen (Grand Island, NY, USA) according to the manufacturer’s instructions. At 48 h post-transfection, transfected cells were selected using 0.8 mg/mL G418 in the media for 3 to 4 weeks. The G418-resistant clones were isolated and screened by measuring their basal and inducible (obtained by treatment with 10 umol tBHQ) luciferase activities as described above. Positive clones, which showed low background and high inducible luciferase activity, were passaged and maintained in growth medium containing 0.8 mg/mL G418.

### Cell viability assay

Cell viability was measured by MTT assay with Cell Counting Kit-8 (Dojindo). 100 μL of cell suspensions (1 × 10^4^ cells/mL) per well were seeded in 96-well plates, incubated at 37 °C and allowed for attachment for 12 h before treatment. Then 10 μL medium per well containing the indicated compounds were added to the wells. Wells containing 110 μL medium without cells were set as a blank control, and experiment control cells were treated with 10 μL medium containing 0.1% DMSO. After certain periods of incubation, 10 μL CCK-8 was added per well. Plates were incubated at 37 °C and 5% CO_2_ for 3 h. Then the optical density (OD) was recorded by ParadigmTM detection platform (Beckman Coulter) at 450 nm. Four wells per dose were conducted in three independent experiments.

### Western blot

HKC cells, seeded in a 6-well plate (6 × 10^5^), were exposed to the indicated compounds for indicated times and harvested by scrapping. Protein concentration of cell lysates was measured by Bradford method. Equal amount of proteins were loaded in sodium dodecyl sulfate-polyacrylamide gels. After electrophoresis, gels were transferred to nitrocellulose membranes. The membranes were washed with PBS containing 0.1% Tween 20 (PBST) thrice 10 min each. Membranes were blocked with 5% nonfat powdered milk for 1 h. Then membranes were incubated with specific primary antibodies overnight at 4 °C, washed with PBST thrice 10 min each, and further incubated with anti-rabbit IgG-HRP conjugates secondary antibodies for 3 h at room temperature. Finally, the blots were visualized using enhanced chemiluminescence (ECL) kit (GE Healthcare).

### Nuclear protein extraction

The extraction and isolation of nuclear and cytoplasmic proteins were performed according to the protocol of Nuclear and Cytoplasmic Protein Extraction Kit (Sangon Biotech). HeLa cells were centrifuged for 5 min at 1000 g at 4 °C and the pellet was dissolved with cytoplasmic protein extraction agent A supplemented with PMSF. After vortex for 15 s, the tubes were incubated for 10–15 min on ice to promote lysis. Next, add the cytoplasmic protein extraction agent B, vortex for 5 s and incubated on ice for 1 min. Vortex for 5 s again, then the samples were centrifuged for 5 min at 14,000 g at 4 °C and the supernatant, consisting of the cytosolic fraction, was immediately frozen for further analysis. The pellet was resuspended in nuclear protein extraction agent supplemented with PMSF. After vertexing the tubes 15–20 times for 30 min and centrifuging for 5 min at 14,000 g, the supernatants containing the nuclear extracts were obtained.

### Pull-down and MS analysis of **J19**-1 interacting proteins

HKC cells were plated on 10 cm dishes and grown to confluence for 1 day. The cells were transfected with Flag-Keap1 for 24 h, and then incubated with Probes or NC for 5 h. Then harvested the cells and incubated with 80 μL streptavidin agarose (Invitrogen) overnight at 4 °C. The beads were washed six times with lysis buffer, then the immunoprecipitations were eluted off the beads using low pH elution buffer at room temperature for 25 min. Acid elution was neutralized by adding 1/20 volume of 1 M Tris-HCl, pH 9.4, and finally boiled in SDS loading buffer. The bead-bound proteins were separated by SDS-PAGE and visualized by silver staining. The protein-containing band in the gel was excised, followed by in-gel digestion and analysis by LC-MS/MS.

### *In vitro* binding analysis

2 μg of WT-Flag-tagged Keap1 or mutant form recombinant proteins were immobilized on flag affinity beads at 4 °C overnight and then incubated with **probe** or **NC** at the indicated concentrations. The mixtures were blotted for biotin or flag.

### Preparation of the recombinant wild-type and the site-mutated Keap1

Site-directed mutagenesis was performed with the QuikChange® site-directed mutagenesis kit (Stratagene) using Flag Keap1 as a template. These proteins were expressed in 293 T cells and purified.

### *In Vivo* Ubiquitination Assay

*In vivo* ubiquitination assay was performed based on the previously reported method^23^. HEK293 cells in a six-well plate for *in vivo* ubiquitination of endogenous Nrf2, HEK293 cells in 100-mm dishes were transfected with the plasmids encoding Keap1, Gal4-tagged Neh2 (or Flag-tagged Nrf2), and HA-tagged ubiquitin. After washing with cold PBS, cells were treated with the indicated compounds for 4 h, and then the cells were lysed and boiled, and were subjected to immunoprecipitation with the indicated antibodies.

### Measurement of intracellular ROS generation

Intracellular ROS were detected using an oxidation-sensitive fluorescent probe 2′, 7′-dichlorodihydrofluorescein diacetate (DCFH-DA). HKC cells were treated with indicated compounds and then washed twice in PBS and placed in a incubator with 2.5 μg/ml DCFH-DA (Sigma-Aldrich) in PBS for 30 min. Subsequently, the cells were washed twice in culture medium and trypsinized. The cell pellet was re-suspended in PBS followed by analysis on a FACSCalibur flow cytometer (Becton-Dickinson).

### siRNA transfections

HKC cells were transfected with Nrf2 small interfering RNA (siRNA) or control siRNA (QIAGEN) by using LipofectAmine 2000 (Invitrogen) for 24 h according to the manufacturer’s protocols (Invitrogen). After 24 h transfection, the medium was removed and replaced for DMEM containing 10% fetal bovine serum. At dedicated time points after transfection, cells were used for MTT cell viability assays or western blot. The siRNA sequence for human Nrf2 was 5′-AAG AGT ATG AGC TGG AAA AAC-3′.

## Additional Information

**How to cite this article**: Dong, T. *et al.* Pterisolic Acid B is a Nrf2 Activator by Targeting C171 within Keap1-BTB Domain. *Sci. Rep.* **6**, 19231; doi: 10.1038/srep19231 (2016).

## Supplementary Material

Supplementary Information
