# Supplementary material for: Pterisolic Acid B is a Nrf2 Activator by Targeting C171 within Keap1-BTB Domain
Source: Sci Rep. 2016 Jan 13;6:19231. doi: 10.1038/srep19231 (PMC4725373; doi:10.1038/srep19231)
Supplement: Supplementary Information [file srep19231-s1.pdf]

Pterisolic Acid B is a Nrf2 Activator by Targeting C171 within Keap1-BTB Domain

Ting Dong, Weilong Liu, Zhirong Shen, Lin Li, She Chen, Xiaoguang Lei *

Table of Contents	
**Supplementary Table1**	S2
**Supplementary Figures S1-7**	S3-S6
**Syntheses of Chemical Probes**	S7-S12
**^{1}H, ^{13}C NMR spectra**	S13-S18

Supplementary Table1: Primer sequences for RT-qPCR

Primer name	sequence
GAPDH-F	5’- GTGTTCCTACCCCCAATGTGT-3’
GAPDH-R	5’- ATTGTCATACCAGGAAATGAGCTT-3’
NQO1-F	5’-GGG CAA GTC CAT CCC AAC TG-3′
NQO1-R	5’-GCA AGT CAG GGA AGC CTG GA-3′
HO-1-F	5’-CCTTCCCGAACATCGACAGCC-3’
HO-1-R	5’-GCAGCTCCTCAAACAGCTCAA-3’

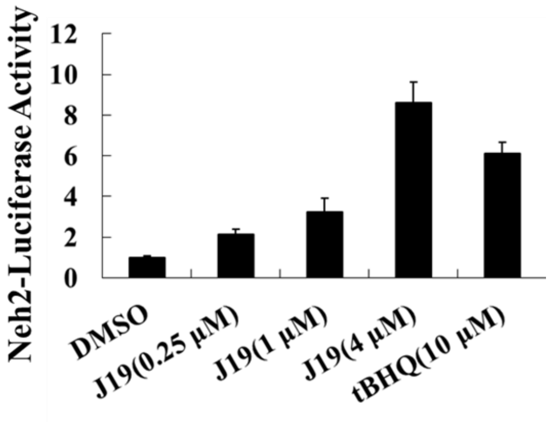

Supplementary Figure S1. J19 induced Nrf2 activation in a time dependent manner. MDA-MB-231-Neh2-Luc stable cell line (N23) cells were treated with **J19** at the indicated concentrations for 24 h, and subject to Luciferase assay. Error bars indicate the standard deviations from triplicate samples.

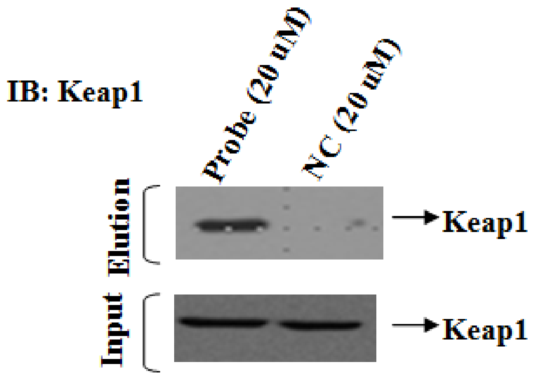

Supplementary Figure S2. J19-1 directly targets Keap1. Total lysates of Keap1-Flag transfected 293T cells were incubated with Probe or NC at 4℃ overnight. The precipitates resolved by SDS-PAGE were analyzed by western blotting.

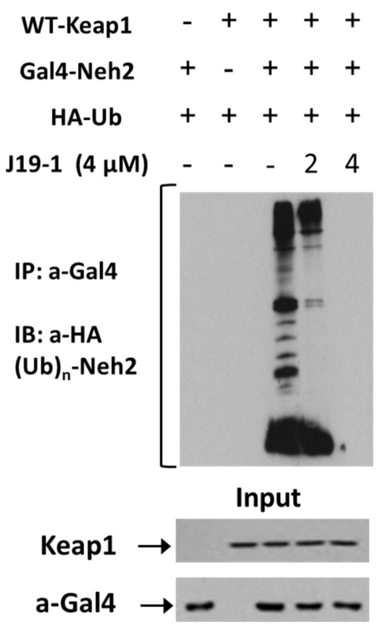

Supplementary Figure S3. J19-1 inhibits Keap1-mediated Nrf2 ubiquitination in a dose dependent manner. HEK293 cells were transiently transfected with WT-Keap1, Gal4-Neh2, and HA-Ub and treated with indicated concentration of **J19-1** or tBHQ for 5 h. Lysates were immunoprecipitated by anti-Gal4 antibody, and ubiquitination was assessed using anti-HA antibody.

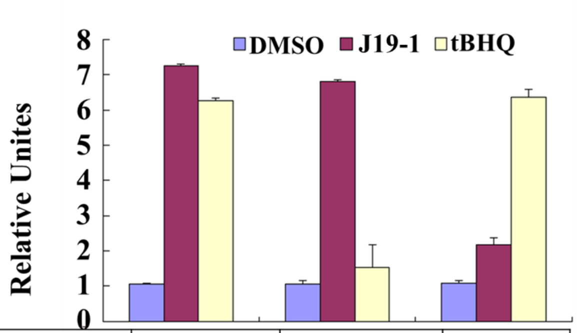

**Nrf2**	+	+	+
**WT-Keap1**	+	-	-
**C151S-Keap1**	-	+	-
**C171S-Keap1**	-	-	+

Supplementary Figure S4. C171S-keap1 behaved identically to the wild-type Keap1 protein in terms of repression of Nrf2-dependent reporter gene activity. 293T cells were transfected with expression vectors for Flag-Nrf2 (100 ng) and mutant

Keap1 proteins (50 ng) as indicated, and with an ARE-dependent firefly luciferase reporter gene construct (100 ng). A plasmid encoding Renilla luciferase (10 ng) was included as a control for transfection efficiency. The data shown represent the means and standard deviation of results from three independent experiments.

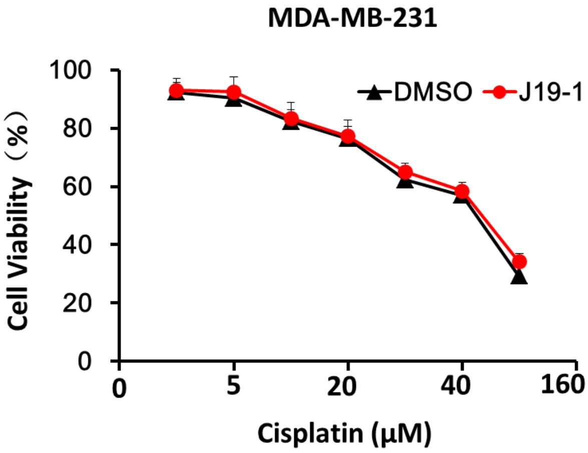

Supplementary Figure S5. Effects of **J19-1** on cisplatin-induced cytotoxicity. MDA-MB231 cells were pretreated with 4 μM of J19-1 for 12 h, followed by exposure to indicated concentration of cisplatin for 48 h. Cell viability was determined using MTT method.

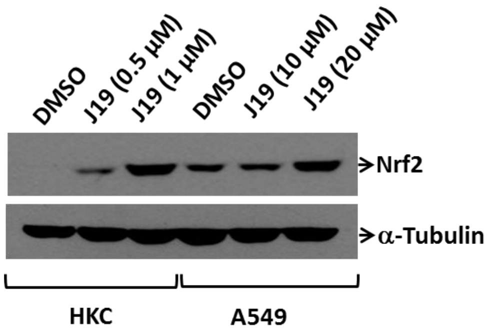

Supplementary Figure S6. Effects of **J19-1** on Nrf2 activation in both normal and cancer cells. HKC and A549 cells were treated with **J19-1** for 4.5h, then the total lysates were subject to immunoblot analysis with anti-Nrf2 and anti-α-Tubulin antibodies.

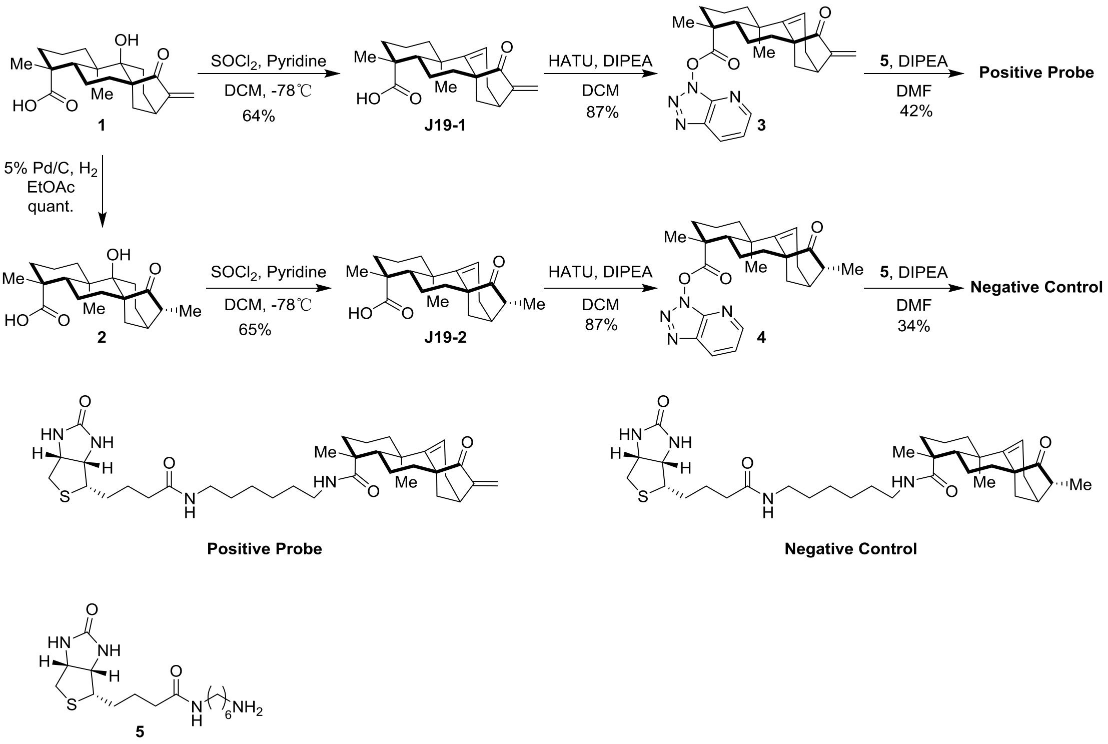

Supplementary Figure S7. Syntheses of **J19** (pterisolic acid B) derivatives and chemical probes

General information:

^{1}H NMR spectra were recorded on a Varian 400 MHz spectrometer at ambient temperature with $CDCl_3$ as the solvent unless otherwise stated. ^{13}C NMR spectra were recorded on a Varian 100 MHz spectrometer (with complete proton decoupling) at ambient temperature. Chemical shifts are reported in parts per million relative to chloroform (^{1}H, δ 7.26; ^{13}C, δ 77.00). Data for ^{1}H NMR are reported as follows: chemical shift, multiplicity (s = singlet, d = doublet, t = triplet, q = quartet, m = multiplet), integration and coupling constants. Infrared spectra were recorded on a Thermo Fisher FT-IR200 spectrophotometer. High-resolution mass spectra were obtained at Peking University Mass Spectrometry Laboratory using a Bruker APEX Flash chromatography. Optical rotations were recorded on an AUTOPOL III digital polarimeter at 589 nm and are recorded as $[\alpha]_D^T$ (concentration in grams/100 mL solvent). Flash chromatography was performed using 200-400 mesh silica gel. Yields refer to chromatographically and spectroscopically pure materials, unless otherwise stated. All reagents were used as supplied by Sigma-Aldrich, J&K and Alfa Aesar Chemicals. *Ent*-9-Hydroxy-15-oxokauran-19-oic acid (**1**) was purchased from BioBioPha Co., Ltd. All reactions were carried out in oven-dried glassware under an argon atmosphere unless otherwise noted.

Detailed experimental procedures:

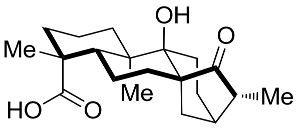

***Ent*-9-Hydroxy-15-oxokauran-19-oic acid (2)**: To a solution of compound **1** (4.9 mg, 0.014 mmol) in ethyl acetate (1 mL) was added Pd/C (5% on carbon, 3 mg) under argon. Then the argon was displaced with hydrogen. The mixture was stirred at room temperature for overnight. After filtration through a short pad of *Celite*, the solution was evaporated *in vacuo* to give 4.9 mg (quant.) of compound **2**[1] as a colorless solid.

1 The characterization data was report by Wang F, Li YJ, Ren FC, Wei GZ, Liu JK. Pterisolic Acids A—F, New ent-Kaurane Diterpenoids from the Fern Pteris semipinnata. *Chem Pharm Bull* 2011; **59**:484-487.

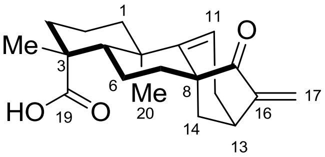

6-Dehydroxyl-pterisolic acid B (J19-1): To a solution of compound **1** (4.8 mg, 0.014 mmol, 1 eq) in 0.5 mL of anhydrous dichloromethane was added a pyridine solution (0.1 mL, 58 μL in 1 mL of anhydrous dichloromethane, 5 eq) at -78℃. Then thionyl chloride (0.1 mL, 21 μL in 1 mL of anhydrous dichloromethane, 2 eq) was added dropwise. The mixture was stirred at -78℃ for 2 h. Then 5 mL of ice-water was added. The mixture was warmed to room temperature and extracted with dichloromethane (15 mL×3). The organic extracts were combined and washed with 1 M HCl (2 mL), brine (5 mL). After dried over Na_2SO_4, the solution was concentrated *in vacuo* and purified by flash chromatography (silica gel, petroleum ether: EtOAc = 4:1) to afford 2.9 mg (64% yield) of compound **J19-1** as waxy solid. $[\alpha]_D^{18.8}$ = +77.8 (c = 0.23, MeOH); ^{1}H NMR (400 MHz $CDCl_3$): δ 5.91 (br s, 1H, H-17), 5.52 (dd, *J* = 3.6 Hz, 3.2 Hz, 1H, H-11), 5.44 (br s, 1H, H-17), 2.95(br s, 1H, H-13), 2.63(ddd, *J* = 17.2 Hz, 4.4 Hz, 3.2 Hz, 1H, H-12), 2.23(m, 1H, H-6), 2.13-2.11(m, 3H, H-12, H-6, H-3), 1.96(m, 1H, H-7), 1.84(m, 1H, H-2), 1.82(m, 1H, H-1), 1.78(m, 1H, H-5), 1.75(m, 1H, H-7), 1.69(m, 2H, H-14), 1.45(m, 1H, H-2), 1.31(s, 3H, H-18), 1.18(m, 1H, H-1), 1.05(s, 3H, H-20), 1.04(m, 1H, H-3); ^{13}C NMR (100 MHz $CDCl_3$): δ 203.2 (C-15), 183.9 (C-19), 151.3 (C-16), 149.5 (C-9), 120.6 (C-11), 116.0 (C-17), 49.5 (C-8), 47.0 (C-5), 44.2 (C-4), 41.3 (C-1), 40.1 (C-14), 39.6 (C-10), 37.5 (C-3), 36.3 (C-13), 36.2 (C-12), 28.4 (C-18), 25.6 (C-7), 22.8 (C-20), 20.1 (C-2), 18.4 (C-6); IR (neat) ν_{max} 1724, 1693, 1463, 1260, 1020, 952 cm^{-1}; HRMS (ESI): $[M-H]^-$ calculated for $C_{20}H_{25}O_3$: 313.1800, found: 313.1797.

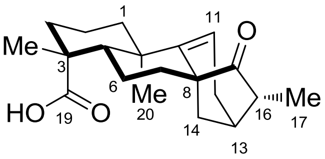

6-Dehydroxyl-16,17-dihydro-pterisolic acid B (J19-2): To a solution of compound **2** (4.9 mg, 0.015 mmol, 1 eq) in 0.5 mL of anhydrous dichloromethane was added a pyridine solution (0.1 mL, 59 μL in 1 mL of anhydrous

dichloromethane, 5 eq) at -78℃. Then thionyl chloride (0.1 mL, 21 μL in 1 mL of anhydrous dichloromethane, 2 eq) was added dropwise. The mixture was stirred at -78℃ for 2 h. Then 5 mL of ice-water was added. The mixture was warmed to room temperature and extracted with dichloromethane (15 mL×3). The organic extracts were combined and washed with 1 M HCl (2 mL), brine (5 mL). After dried over Na_2SO_4, the solution was concentrated *in vacuo* and purified by flash chromatography (silica gel, petroleum ether: EtOAc = 4:1) to afford 3 mg (65% yield) of compound **J19-2** as colorless solid. $[\alpha]_D^{18.0}$= +216.6 (c = 0.18, MeOH); ^{1}H NMR (400 MHz $CDCl_3$): δ 10.72 (br s, -COOH), 5.48 (br s, 1H, H-11), 2.38 (m, 1H, H-13), 2.33 (m, 2H, H-16,H-12), 2.14 (m, 1H, H-12), 2.13 (m, 1H, H-6), 2.10 (m, 1H, H-3), 2.02 (m, 1H, H-6), 1.88 (m, 1H, H-7), 1.82 (m, 1H, H-2), 1.81 (m, 1H, H-5), 1.78 (m, 1H, H-1), 1.73 (m, 2H, H-14), 1.71 (m, 1H, H-7), 1.44 (m, 1H, H-2), 1.29 (s, 3H, H-18), 1.14 (m, 1H, H-1), 1.12 (d, *J* = 7.2 Hz, 3H, H-17), 1.05 (m, 1H, H-3), 1.01 (s, 3H, H-20); ^{13}C NMR (100 MHz $CDCl_3$): δ 216.3 (C-15), 183.8 (C-19), 148.6 (C-9), 120.9 (C-11), 49.3 (C-8), 47.8 (C-16), 47.3 (C-5), 44.1 (C-4), 41.5 (C-1), 41.3 (C-14), 39.4 (C-10), 37.3 (C-3), 33.0 (C-13), 28.3 (C-18), 28.0 (C-12), 26.4 (C-7), 22.6 (C-20), 20.2 (C-2), 18.7 (C-6), 12.9 (C-17); IR (neat) ν_{max} 1728, 1691, 1452, 1260, 1088, 1017, 939 cm^{-1}; HRMS (ESI): $[M+Na]^+$ calculated for $C_{20}H_{28}NaO_3$: 339.1931, found: 339.1947.

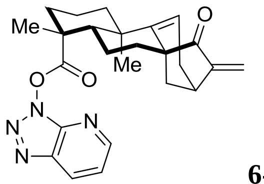

6-Dehydroxyl-pterisolic acid B active ester (3): Compound **J19-1** (2.9 mg, 0.009 mmol) and HATU (7 mg, 0.018 mmol) were dissolved in 1 mL of anhydrous dichloromethane, then N, N-diisopropylethylamine (15 μL, 0.092 mmol) was added. After 2.5 h, the solution was concentrated *in vacuo* and purified by flash chromatography (silica gel, petroleum ether: EtOAc = 5:1) to afford 3.5 mg (87% yield) of compound **3** as a waxy solid. $[\alpha]_D^{20.2}$= +112.0 (c = 0.15, $CHCl_3$); ^{1}H NMR (400 MHz $CDCl_3$): δ 8.71(d, *J* = 4.4 Hz, 1H), 8.40(d, *J* = 8.4 Hz, 1H), 7.42(dd, *J* = 8.4 Hz, 4.4 Hz, 1H), 5.94(br s, 1H), 5.58(br s, 1H), 5.47(br s, 1H), 2.98(br s, 1H), 2.65(br d, *J* = 17.6 Hz,

1H), 2.46(m, 1H), 2.42(m, 1H), 2.25(m, 1H), 2.15(br d, J = 17.6 Hz, 1H), 1.99-1.98(m, 3H), 1.95(m, 1H), 1.84(m, 1H), 1.71(m, 2H), 1.68(s, 3H), 1.62(m, 1H), 1.31(m, 1H), 1.28(m, 1H), 1.23(s, 3H) ; ^{13}C NMR (100 MHz $CDCl_3$): δ 203.3, 173.4, 151.7, 151.3, 149.1, 140.8, 135.0, 129.3, 120.7, 120.6, 116.2, 49.5, 47.2, 45.3, 40.8, 39.9, 39.6, 37.8, 36.3, 36.1, 28.0, 25.1, 23.6, 20.2, 18.5; IR (neat) ν_{max} 1817, 1797, 1721, 1245, 1123, 1004 cm^{-1}; HRMS (ESI): $[M+H]^+$ calculated for $C_{25}H_{29}N_4O_3$: 433.2234, found: 433.2230.

HATU=1-[Bis(dimethylamino)methylene]-1H-1,2,3-triazolo[4,5-b]pyridinium-3-oxid hexafluorophosphate.

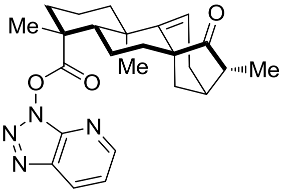

6-Dehydroxyl-16,17-dihydro-pterisolic acid B active ester (4): Compound **J19-2** (3 mg, 0.0095 mmol) and HATU (7 mg, 0.019 mmol) was dissolved in 1 mL of anhydrous dichloromethane, then N, N-Diisopropylethylamine (16 μL, 0.095 mmol) was added. After 2.5 h, the solution was concentrated *in vacuo* and purified by flash chromatography (silica gel, petroleum ether: EtOAc = 5:1) to afford 3.6 mg (87% yield) of compound **4** as a colorless solid. $[\alpha]_D^{21.9}$= +194.3 (c = 0.23, $CHCl_3$); ^{1}H NMR (400 MHz $CDCl_3$): δ 8.70 (d, J = 4.4 Hz, 1H), 8.40 (d, J = 8.4 Hz, 1H), 7.41 (dd, J = 8.4 Hz, 4.4 Hz, 1H), 5.40 (br s, 1H), 2.46-2.27 (m, 5H), 2.23-2.12 (m, 2H), 2.05-1.88 (m, 4H), 1.83-1.70(m, 3H), 1.66 (s, 3H), 1.64-1.59 (m, 1H), 1.36-1.23 (m, 2H), 1.20 (s, 3H), 1.14 (d, J = 6.8 Hz, 3H); ^{13}C NMR (100 MHz $CDCl_3$): δ 216.4, 173.5, 151.7, 148.2, 140.8, 135.0, 129.2, 120.9, 120.7, 49.3, 47.9, 47.4, 45.1, 41.1, 41.0, 39.4, 37.6, 33.0, 28.0, 25.9, 23.4, 20.2, 18.8, 12.8; IR (neat) ν_{max} 1797, 1725, 1454, 1244, 1122, 1001 cm^{-1}; HRMS (ESI): $[M+H]^+$ calculated for $C_{25}H_{31}N_4O_3$: 435.2391, found: 435.2388.

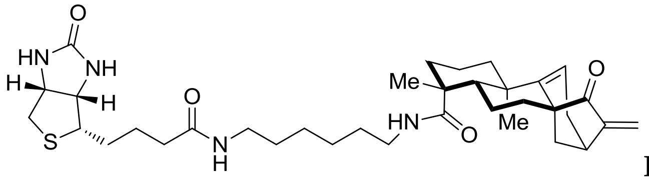

Biotinylated 6-dehydroxyl-pterisolic acid B (Positive Probe): Active ester **3** (3.5 mg, 0.0081 mmol) was dissolved in 0.5 mL

of anhydrous DMF. Then Diisopropylethylamine (40 μL, 0.24 mmol) was added followed by amine **5** (110 μL, 77 mg in 1 mL of anhydrous DMF, 0.024 mmol). The reaction mixture was warmed to 60℃ and stirred for overnight. The solution was diluted with dichloromethane (10 mL), washed with H_2O (5 mL), brine (5 mL×2). The organic phase was dried over anhydrous Na_2SO_4, concentrated and purified by flash chromatography (silica gel, dichloromethane: methanol=10:1) to afford 2.1 mg (42% yield) of **positive probe** as a waxy solid. $[\alpha]_D^{21.1}$ = +108.0 (c = 0.08, MeOH); ^{1}H NMR (400 MHz $CDCl_3$): δ 6.05(br s, 1H), 5.91(br s, 1H), 5.82(br, 1H), 5.74(br s, 1H), 5.51(br s, 1H), 5.44(br s, 1H), 5.05(br, 1H), 4.52(m, 1H), 4.33(m, 1H), 3.22(m, 4H), 3.15(m, 1H), 2.94(br s, 1H), 2.92(dd, *J* = 12.4 Hz, 4.4 Hz, 1H), 2.74(d, *J* = 12.4 Hz, 1H), 2.62(br d, *J* = 17.6 Hz, 1H), 2.22 (t, *J* = 7.2 Hz, 2H), 2.19(m, 1H), 2.11(br d, *J* = 17.6 Hz, 1H), 2.02(m, 1H), 2.01(m, 1H), 1.94(m, 1H), 1.84(m, 1H), 1.79(m, 1H), 1.73(m, 1H), 1.71-1.69(m, 7H), 1.49-1.45(m, 5H), 1.35-1.33(m, 4H), 1.25(m, 2H), 1.24(s, 3H), 1.18(m, 1H), 1.15(m, 1H), 1.05(s, 3H). ^{13}C NMR (100 MHz $CDCl_3$): δ 203.1, 177.3, 173.0, 163.3, 151.2, 149.5, 121.0, 116.1, 61.7, 60.1, 55.4, 49.5, 47.8, 43.9, 41.9, 40.5, 40.2, 39.7, 39.0, 39.0, 38.0, 36.3, 36.1, 35.9, 29.7, 29.6, 29.4, 28.0, 26.4, 26.2, 26.0, 25.6, 22.8, 20.5, 19.4; IR (neat) ν_{max} 3296, 2924, 2853, 1703, 1641, 1529, 1462, 1262 cm^{-1}; HRMS (ESI): $[M+H]^+$ calculated for $C_{36}H_{55}N_4O_4S$:639.3939, found:639.3954.

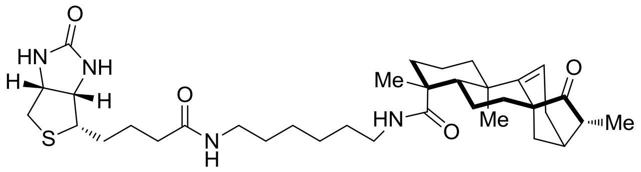

Biotinylated 6-dehydroxyl-16, 17-dihydro-pterisolic acid B (Negative Control): Active ester **4** (3.6 mg, 0.0083 mmol) was dissolved in 0.5 mL of anhydrous DMF. Then Diisopropylethylamine (41 μL, 0.25 mmol) was added followed by amine **5** (110 μL, 77 mg in 1 mL of anhydrous DMF, 0.025 mmol). The reaction mixture was warmed to 60℃ and stirred for overnight. The solution was diluted with dichloromethane (10 mL), washed with H_2O (5 mL), brine (5 mL×2). The organic phase was dried over anhydrous Na_2SO_4, concentrated and purified by flash chromatography (silica gel, dichloromethane: methanol=10:1) to afford 1.7 mg

(34% yield) of **negative control** as a colorless solid. $[\alpha]_D^{21.4}$ = +100.0 (c=0.07, MeOH); ^{1}H NMR (400 MHz $CDCl_3$): δ 5.97 (br s, 1H), 5.71 (br s, 1H), 5.61 (br, 1H), 5.48 (br s, 1H), 4.91 (br, 1H), 4.52 (m, 1H), 4.33 (m, 1H), 3.21 (m, 4H), 3.17 (m, 1H), 2.92 (dd, *J* = 12.8 Hz, 4.8Hz, 1H), 2.74 (d, *J* = 12.8 Hz, 1H), 2.38 (m, 1H), 2.33 (m, 1H), 2.32 (m, 1H), 2.21 (m, 2H), 2.12 (m, 1H), 2.11 (m, 1H), 1.95 (m, 1H), 1.93 (m, 2H), 1.81 (m, 2H), 1.75 (m, 1H),1.74 (m, 1H),1.70-1.69 (m, 6H), 1.49-1.45 (m, 5H) , 1.34-1.33 (m, 4H), 1.25(m, 2H), 1.22 (s, 3H), 1.16 (m, 1H), 1.15 (m, 1H), 1.12 (d, *J* = 6.8 Hz, 3H), 1.00 (s, 3H); ^{13}C NMR (100 MHz $CDCl_3$): δ 216.1, 177.4, 172.9, 163.1, 148.5, 121.5, 61.7, 60.1, 55.3, 49.3, 48.2, 47.6, 43.9, 42.2, 41.5, 40.6, 39.6, 39.0, 38.9, 37.8, 35.9, 33.0, 30.9, 29.6, 29.4, 28.03, 27.98, 27.4, 26.2, 26.0, 25.6, 22.6, 20.5, 19.7, 13.0; IR (neat) ν_{max} 3286, 2925, 2855, 1700, 1637, 1524, 1456, 1261 cm^{-1}; HRMS (ESI): $[M+H]^+$ calculated for $C_{36}H_{57}N_4O_4S$:641.4095, found:641.4109.

^{1}H, ^{13}C NMR spectra

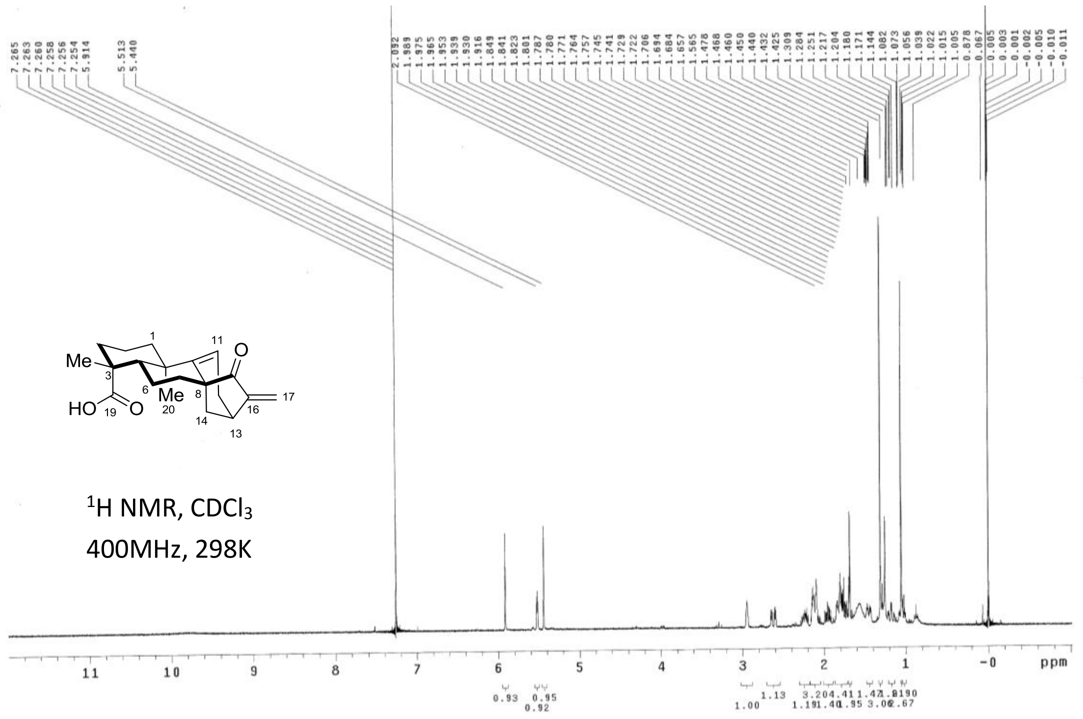

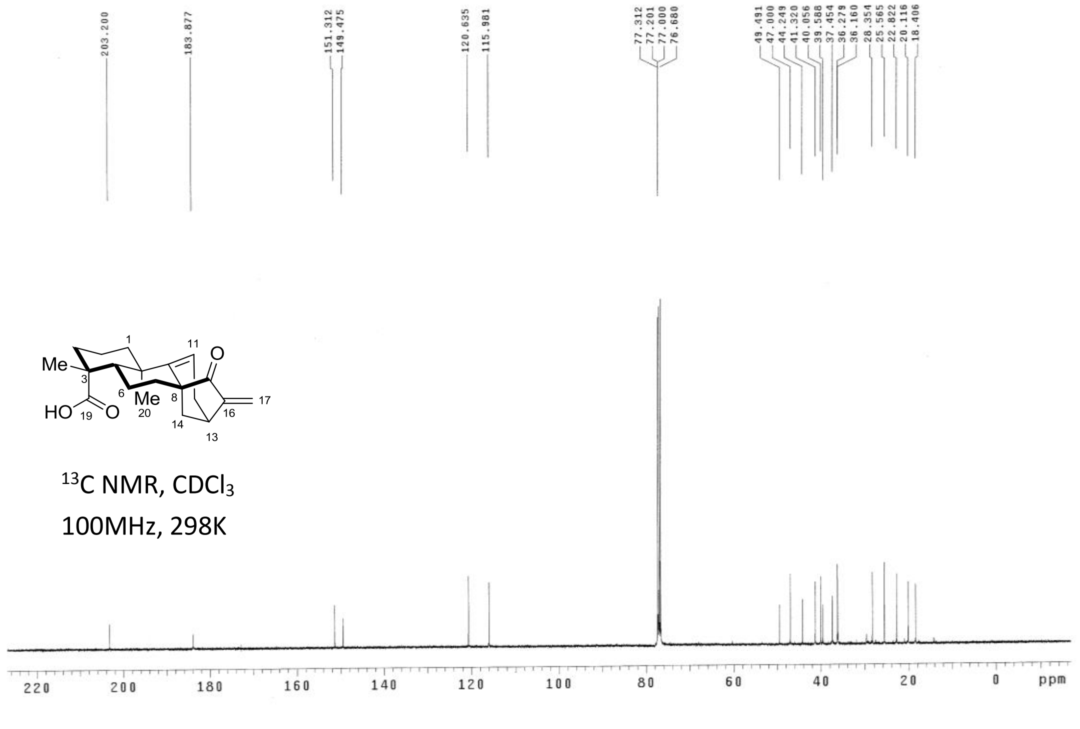

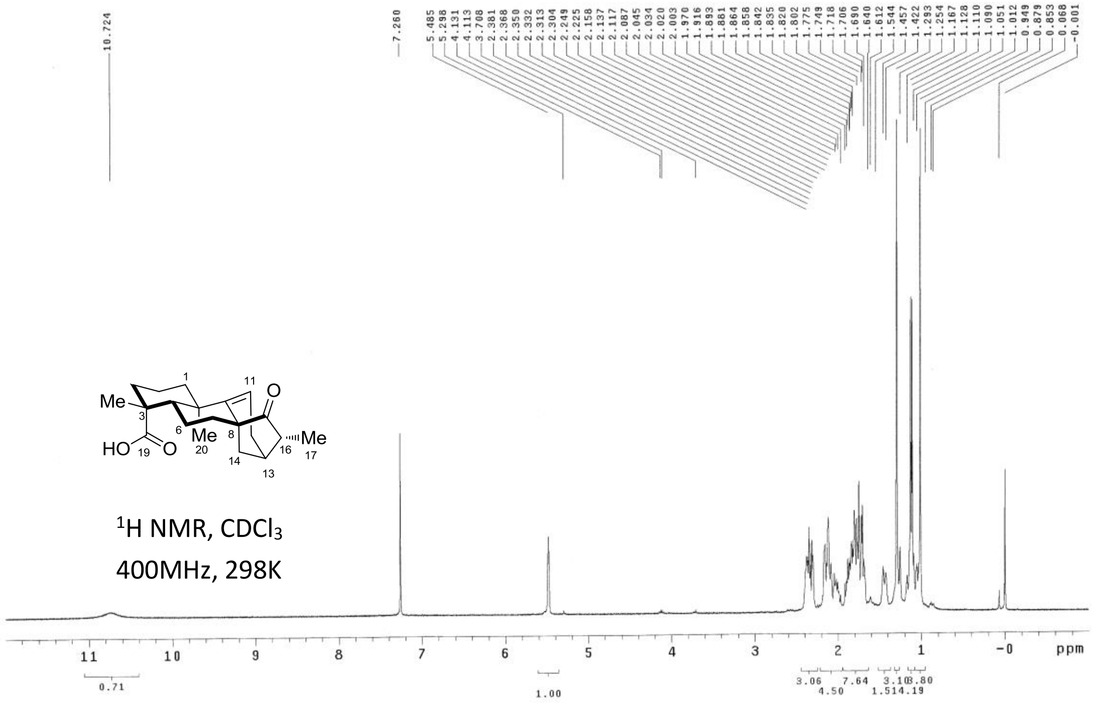
Me
HO
O
O
Me
Me
1
3
6
8
11
13
14
16
17
19
20
^{1}H NMR, $CDCl_3$
400MHz, 298K
ppm

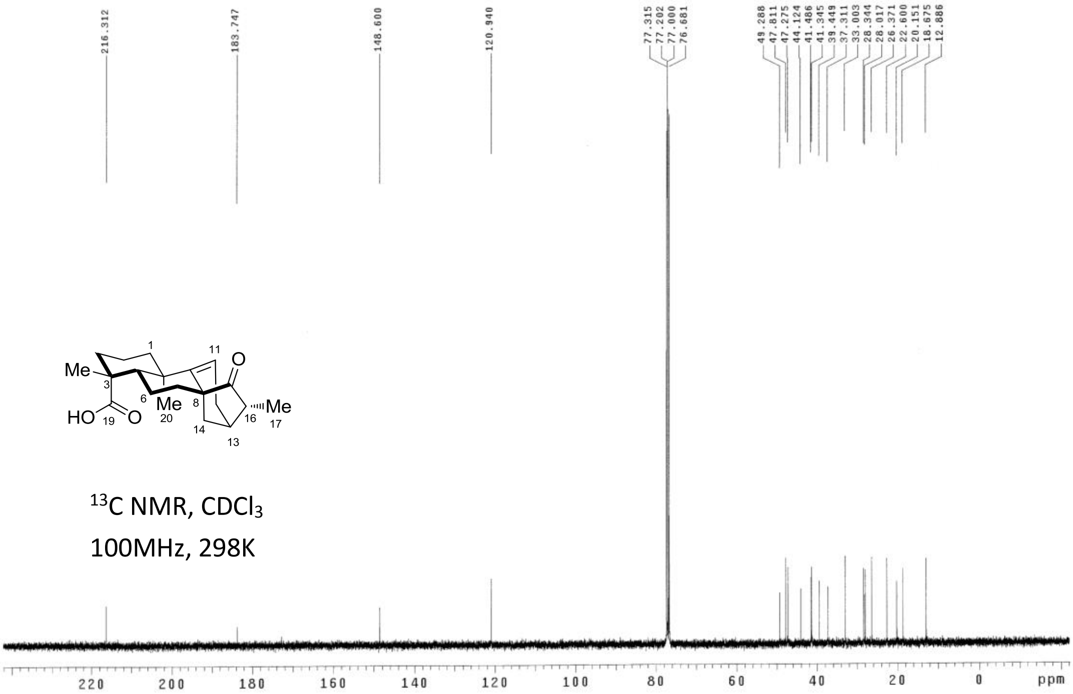
Me
HO
O
O
Me
Me
1
3
6
8
11
13
14
16
17
19
20
^{13}C NMR, $CDCl_3$
100MHz, 298K
ppm

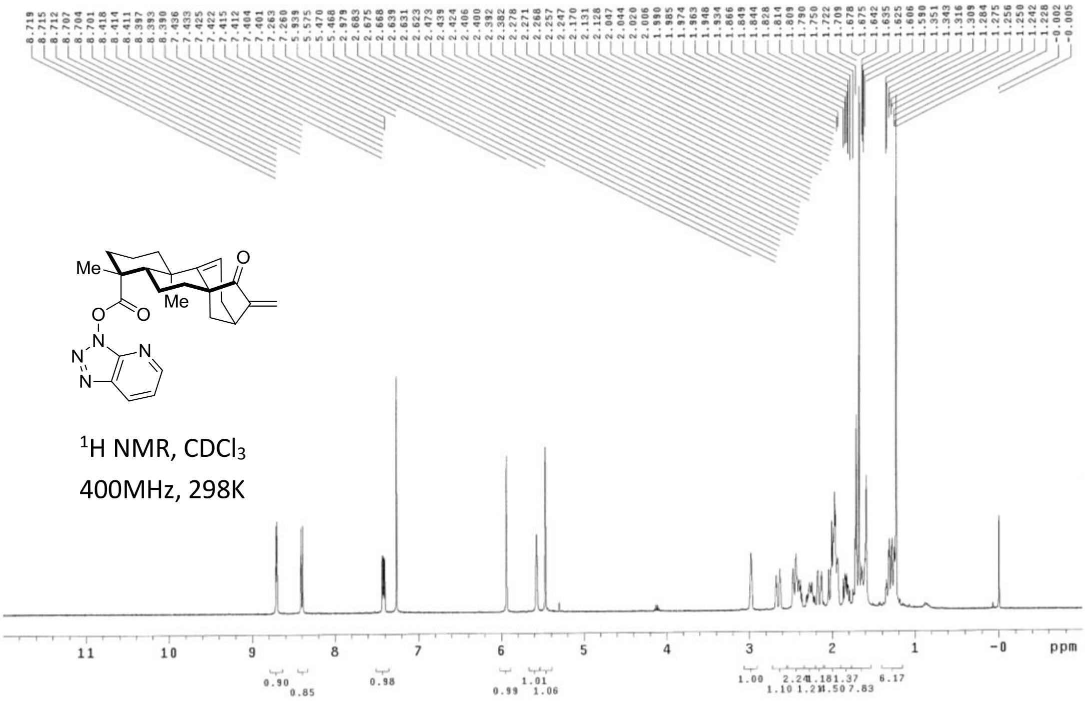

Me
Me
O
O
O
N
N
N
N
N
^{1}H NMR, $CDCl_3$
400MHz, 298K
ppm

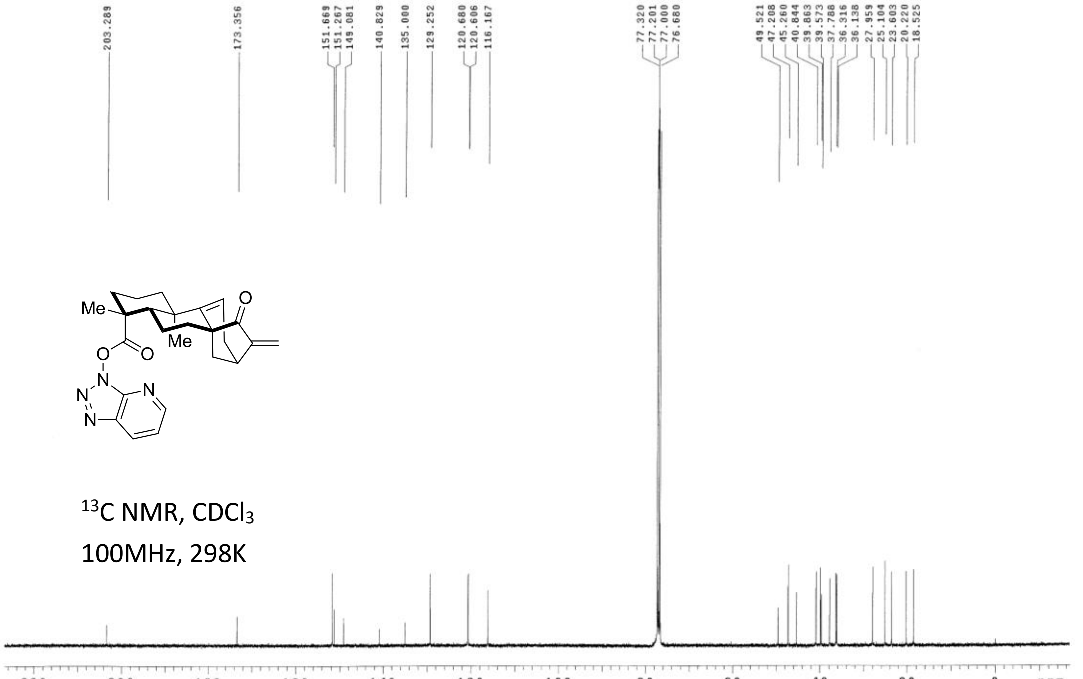

Me
Me
O
O
O
N
N
N
N
N
^{13}C NMR, $CDCl_3$
100MHz, 298K
ppm

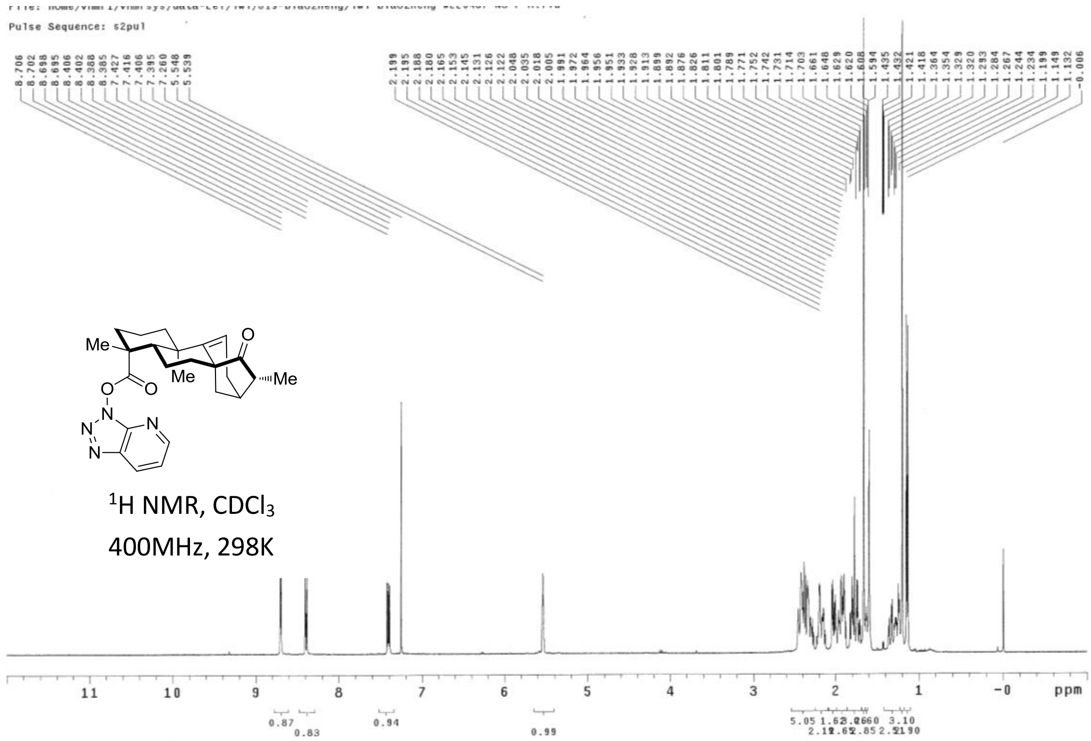
Pulse Sequence: s2pul
Me
O
O
Me
Me
O
N
N
N
N
N
1H NMR, CDCl3
400MHz, 298K
ppm

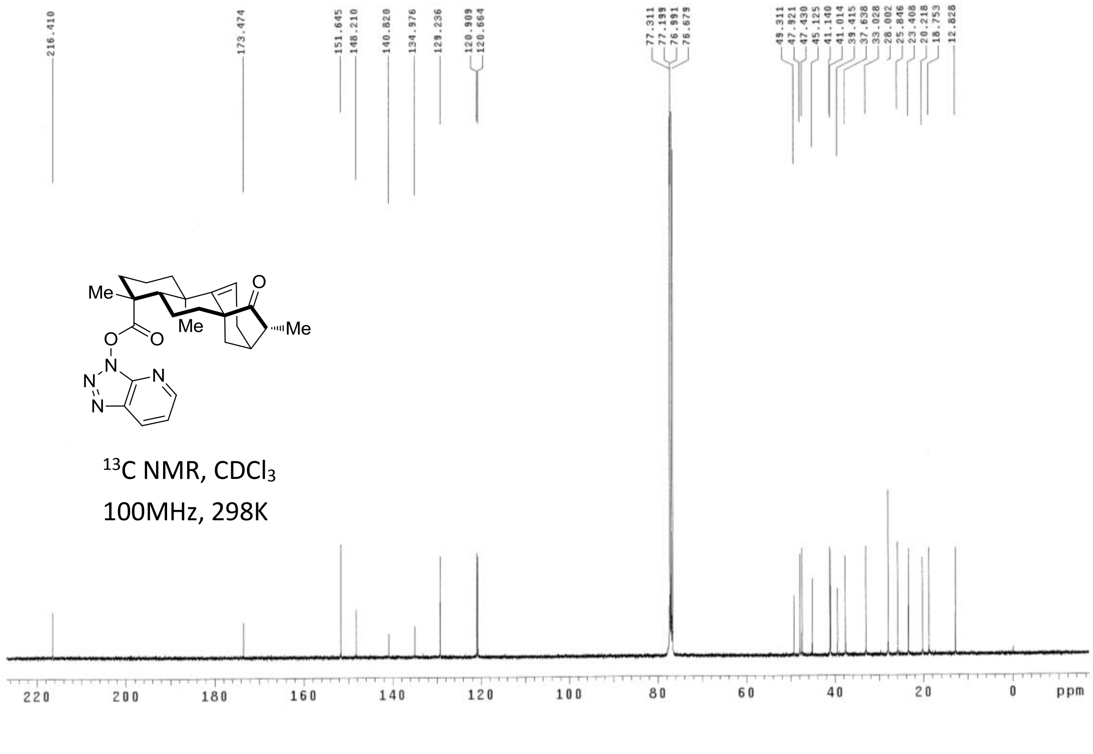
216.410
173.474
151.645
148.210
140.820
134.976
129.236
120.909
120.664
77.311
77.199
76.991
76.679
49.311
47.921
47.430
45.125
41.140
41.014
39.415
37.638
33.028
28.002
25.846
23.408
20.218
18.753
12.828
Me
O
O
Me
Me
O
N
N
N
N
N
13C NMR, CDCl3
100MHz, 298K
ppm

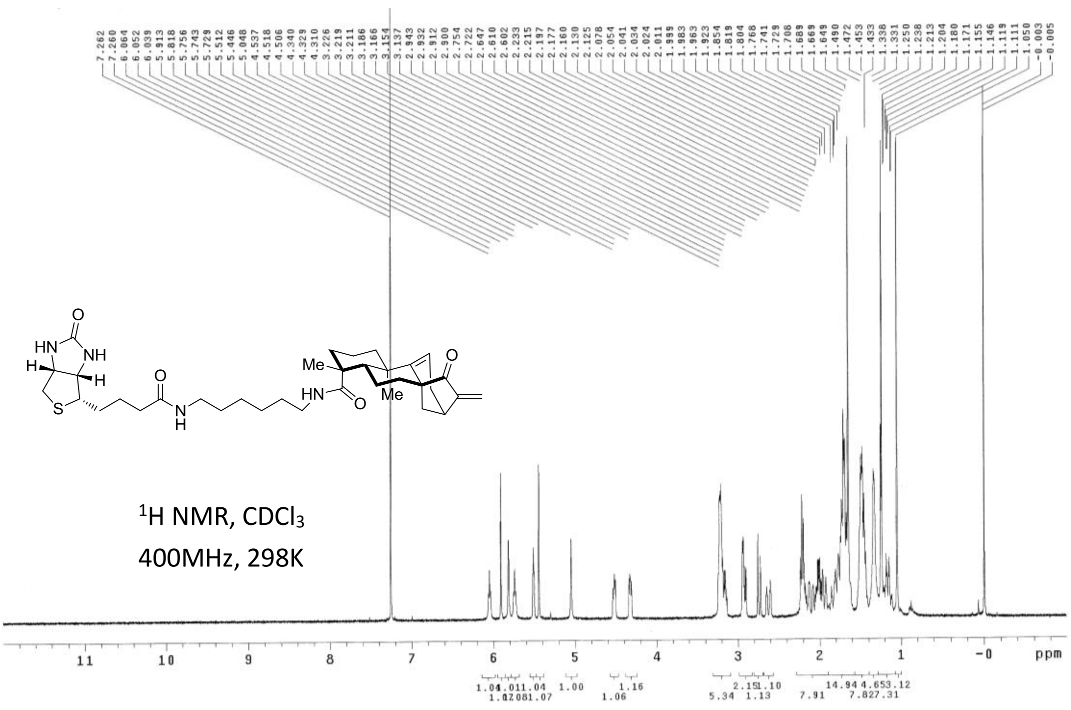

^{1}H NMR, $CDCl_3$
400MHz, 298K
ppm

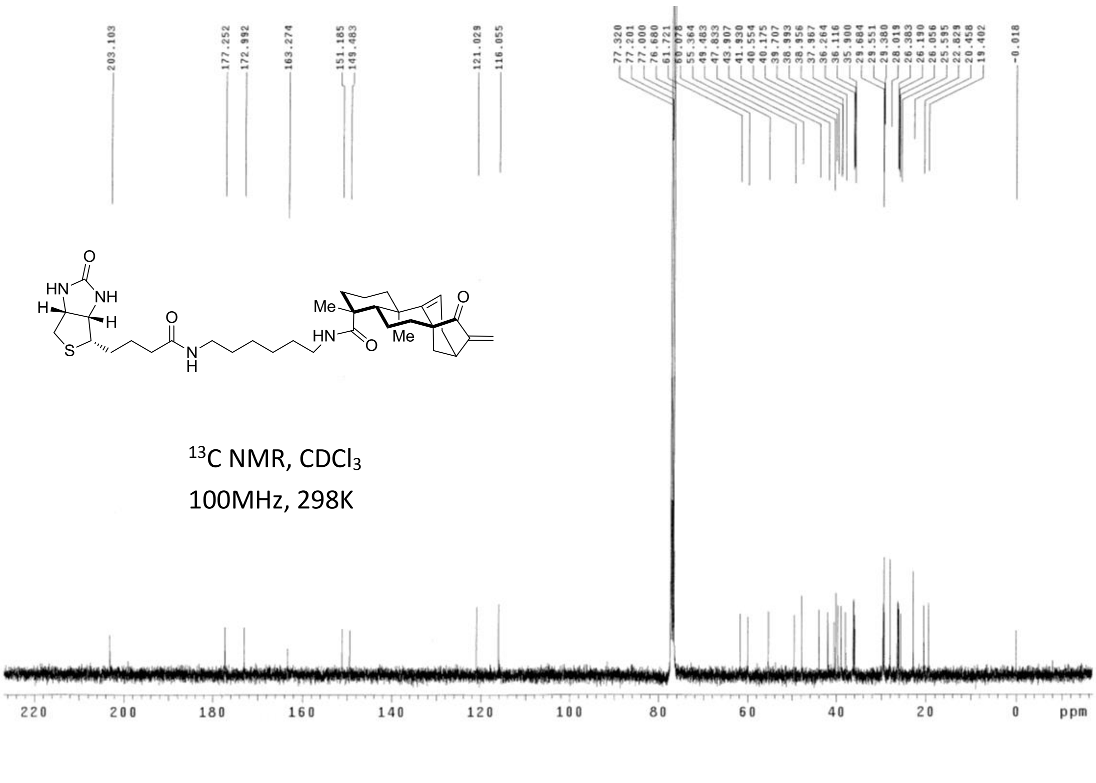

^{13}C NMR, $CDCl_3$
100MHz, 298K
ppm

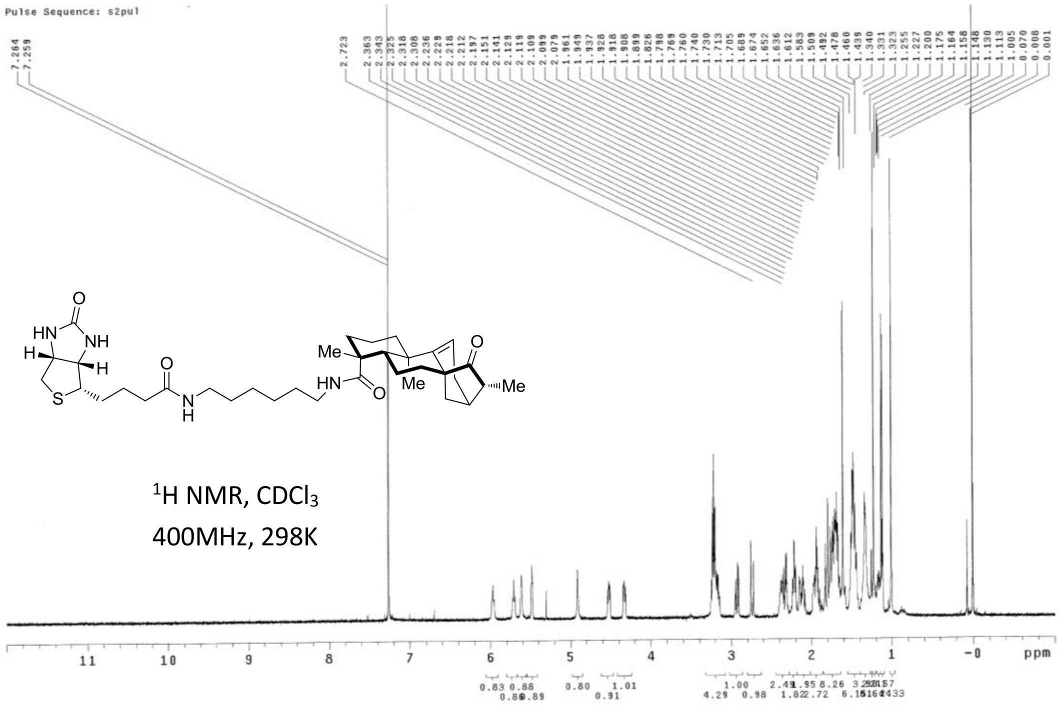
Pulse Sequence: s2pul
HN
NH
H
H
S
O
N
H
HN
O
Me
Me
Me
O
^{1}H NMR, $CDCl_3$
400MHz, 298K
ppm

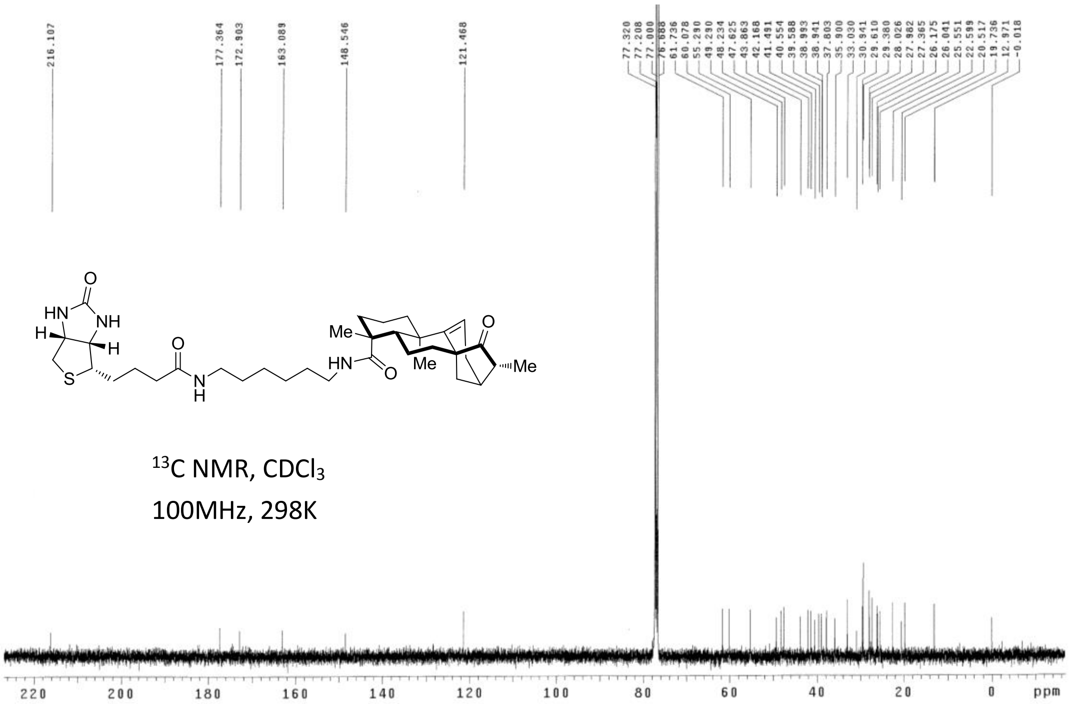
HN
NH
H
H
S
O
N
H
HN
O
Me
Me
Me
O
^{13}C NMR, $CDCl_3$
100MHz, 298K
ppm
